# Low Diversity of Human Milk Oligosaccharides is Associated with Necrotising Enterocolitis in Extremely Low Birth Weight Infants

**DOI:** 10.3390/nu10101556

**Published:** 2018-10-20

**Authors:** Erik Wejryd, Magalí Martí, Giovanna Marchini, Anna Werme, Baldvin Jonsson, Eva Landberg, Thomas R. Abrahamsson

**Affiliations:** 1 Department of Clinical and Experimental Medicine, Linköping University, 58183 Linköping, Sweden; erik.wejryd@liu.se (E.W.); magali.marti.genero@liu.se (M.M.); anna.werme@gmail.com (A.W.); eva.landberg@regionostergotland.se (E.L.); 2 Department of Neonatology, Karolinska University Hospital, 17176 Stockholm, Sweden; giovanna.marchini@sll.se (G.M.); baldvin.jonsson@sll.se (B.J.); 3 Department of Women´s and Children´s Health, Karolinska Insitute, 17177 Stockholm, Sweden; 4 Department of Clinical Chemistry, Linköping University, 58185 Linköping, Sweden; 5 Department of Pediatrics, Linköping University, 58183 Linköping, Sweden

**Keywords:** neonatal, preterm, breast milk, oligosaccharides, diversity, necrotizing enterocolitis, sepsis, growth

## Abstract

Difference in human milk oligosaccharides (HMO) composition in breast milk may be one explanation why some preterm infants develop necrotizing enterocolitis (NEC) despite being fed exclusively with breast milk. The aim of this study was to measure the concentration of 15 dominant HMOs in breast milk during the neonatal period and investigate how their levels correlated to NEC, sepsis, and growth in extremely low birth weight (ELBW; <1000 g) infants who were exclusively fed with breast milk. Milk was collected from 91 mothers to 106 infants at 14 and 28 days and at postmenstrual week 36. The HMOs were analysed with high-performance anion-exchange chromatography with pulsed amperometric detection. The HMOs diversity and the levels of Lacto-N-difucohexaose I were lower in samples from mothers to NEC cases, as compared to non-NEC cases at all sampling time points. Lacto-N-difucohexaose I is only produced by secretor and Lewis positive mothers. There were also significant but inconsistent associations between 3′-sialyllactose and 6′-sialyllactose and culture-proven sepsis and significant, but weak correlations between several HMOs and growth rate. Our results suggest that the variation in HMO composition in breast milk may be an important factor explaining why exclusively breast milk fed ELBW infants develop NEC.

## 1. Introduction

While the care of premature infants has improved dramatically during the last decades, still about 30% of the extremely low birth weight (ELBW, birth weight < 1000 g) infants die [1]. Severe infections and necrotizing enterocolitis (NEC) are common causes of death in this population, and there are clear links between nutrition and the risk of NEC, infection, and mortality [2]. Definite NEC (Bell’s stage II-III [3]) remains among the most devastating diseases encountered in premature infants. It is associated with an excessive inflammatory process in the intestinal mucosa that presents clinically with feeding intolerance, abdominal distension, and bloody stools [4]. The incidence among ELBW infants is approximately 10% [5], but varies in different neonatal settings between 4 to 15% depending on factors such as breastfeeding rates and use of milk banks [6]. The surgical intervention rate is as high as 50% [4], and the mortality rate in affected infants is 15–30%. It is increasingly recognized that NEC is a major adverse factor for subsequent lower intelligence quotient (IQ), motor impairment, visual impairment, and cerebral palsy [7]. Finding new measures to identify and treat infants at-risk is urgently needed.

Exclusive enteral feeding with human breast milk remains the most important prevention strategy for NEC [2]. However, in Scandinavian countries, despite exclusive feeding with breast milk being employed routinely for at least 20 years, the incidence of NEC in ELBW infants is still as high as 10% according to the most recent data available in the Swedish Neonatal Quality Register (www.snq.se). Breast milk not only provides the necessary nutrients for growth and development, it also contains numerous immunological components that compensate the immature and inexperienced mucosal immune system [8]. Such components include immune cells, IGA antibodies, and pro and anti-inflammatory cytokines such as TNF and IL-10 [8,9].

Difference in the composition of bioactive components in breast milk may explain why some preterm infants still develop NEC despite being fed exclusively with breast milk. Among these, non-digestible human milk oligosaccharides (HMOs) are highly abundant (5–15 g/L) as constituents. Besides stimulating beneficial microbes, such as bifidobacteria, in the infant intestinal tract [10], HMOs mimic carbohydrate-binding motifs of certain enteric pathogens, such as enteropathogenic *E. coli*, by acting as a receptor decoy, and thereby prevent adhesion of these pathogens to the apical surface of enterocytes [11]. Certain HMOs also stimulate anti-inflammatory responses in the intestinal epithelium [12,13]. A growing body of evidence suggests that the HMO composition in breast milk influences the risk of developing NEC in preterm infants [14,15,16]. Supplementation with the HMOs disialyllacto-N-tetraose (DSLNT) [14] and 2′-fucosyllactose (2FL) were identified to be protective against NEC in an experimental murine model. Low DSLNT levels preceded NEC development in very low birth weight preterm infants (VLBW; <1500 g) in a human trial [16]. Interestingly, there was a high variation of DSLNT in different countries in a world-wide study in which Swedish mothers had the lowest levels [17]. HMO levels have also been associated with growth [18] and the risk of infections in infants [19,20].

The aim of this study was to investigate the composition of 15 dominant HMOs in breast milk during the neonatal period and examine how this correlated to NEC, sepsis, and growth in ELBW infants who were exclusively fed with breast milk. The analyses revealed low HMO diversity and low levels of the HMO Lacto-N-difucohexaose (LNDH I) in infants that developed NEC.

## 2. Materials and Methods

### 2.1. Study Design and Participants

The present study was a part of the prospective, randomized-controlled, multi-centre trial “Prophylactic Probiotics to Extremely Low Birth Weight Premature Infants” (PROPEL) evaluating the effect of probiotic *Lactobacillus reuteri* DSM 17938 on feeding tolerance, growth, severe morbidities, and mortality in ELBW premature infants (ClinicalTrials.gov ID NCT01603368). A detailed study design and the clinical outcomes have been published elsewhere [21]. Briefly, in total 134 infants born between gestational week (gw) 23 + 0 and 27 + 6 with a birth weight below 1000 g were enrolled between 2012 and 2015 at two level III neonatal intensive care units (Astrid Lindgren Children’s Hospital, Stockholm, and Linköping University Hospital, Linköping, Sweden). Exclusion criteria were major congenital or chromosomal anomalies, no realistic hope of survival, the infant could not be fed and thus not receive the study product within three days, or the infant was included in another intervention trial on growth, feeding intolerance, or severe morbidity. Participating infants received daily oral administration of 1.25 ×10^8^ *L. reuteri* DSM 17938 or placebo from birth to postmenstrual week 36 + 0. Written informed consent was obtained from both parents and the study was approved by the Ethics Committee for Human Research at Linköping University (Dnr 2012/28-31, Dnr 2012/433-32). Due to the 100% coverage of breast milk donor banks, all infants were fed exclusively with breast milk until they had reached a weight of at least 2000 g. Protein and lipid fortification was based individually on analyses of the macronutrient and energy content of the breast milk given to each infant. Oral feeding started during the first day of life and increased gradually at a rate specified in clinical guidelines. Breast milk fortification with bovine protein fortifier started when the enteral feeds had reached 100 mL/kg/day. Breast milk samples were collected from 91 mothers to 106 infants at 14 (*n* = 78) and 28 (*n* = 71) days after delivery and at postmenstrual week (PMW) 36 + 0 (*n* = 51). The milk was frozen in sterile tubes at −20 °C (short-term) and subsequently at −70 °C. The median time until the analysed breast milk was given to the infant was five days (inter-quartile range [IQR] 1–7). Samples were only obtained if the infant was exclusively fed mother´s own milk. All infants to mothers from whom samples were available were included in the present study. Mother´s own milk was not pasteurised before feeding at the neonatal intensive care units (NICUs) at the time of the trial. Analytic staff was blinded to the clinical metadata until the laboratory analyses were concluded.

### 2.2. Clinical Outcomes

The infants were characterized using comprehensive clinical data including perinatal data, growth, antibiotics, and mild to severe morbidities collected daily in a study specific case report form until gestational week 36 + 0. Necrotizing enterocolitis (NEC) was staged according to Bell’s criteria [3], and all cases of stage II or greater were recorded. A diagnosis of culture-proven sepsis required a positive blood culture, clinical deterioration, and a laboratory inflammatory response. Weight, length and head circumference were recorded at birth, at 14 and 28 days, and at PMW 36 + 0. In order to adjust for gestational age, the standard deviation score (z-score) for each measurement was calculated using Niklasson’s growth chart, which is based on information of normal deliveries from gestational week 24 to full term in the Swedish medical birth registry, 1990–1999 [22]. The growth rate was calculated using the difference in z-score between the later measurements and birth.

### 2.3. Purification of Human Milk Oligosaccharides

HMOs were purified from each milk sample by the following method. To remove lipids, 1 mL of the milk sample was centrifuged at 20,000 *g* for 30 min. Thereafter, 0.5 mL of the infranatant fluid was transferred to another tube and 50 µL of the internal standard was added. The internal standard consisted of 2.4 mg/mL galacturonic acid (Sigma-Aldrich, St Louis, MO, USA) and 12 mg/mL stachyose (Sigma-Aldrich). To precipitate proteins, 1 mL of refrigerated 99.5 % ethanol was added, and the mixture was kept at 4 °C for one hour and subsequently centrifuged at 20,000 *g* for 10 min at 4 °C. The sample was further purified by applying 1 mL of the supernatant fluid to an Isolute C18 column (Biotage, Uppsala, Sweden), preconditioned with 2 mL of ethanol followed by 2 mL of water. The eluate was collected and then ultra-filtrated using Amicon Ultra-4 (Merck Millipore, Cork, Ireland) with a 3 kDa molecular cut off. The tube was centrifuged at 4000 *g* for 40 min and the filtered sample was collected. To remove ethanol, the filtrate was evaporated with compressed air for one hour at 40 °C. Neutral oligosaccharides were further purified by applying 50 µL of the sample to an anion exchange bonded silica cartridge (LC-SAX, Supelco, Bellefonte, PA, USA), preconditioned according to instructions. Thereafter, 500 µL of Milli-Q water was added to the column and the eluate was collected.

### 2.4. Analysis of Human Milk Oligosaccharides

High-performance anion-exchange chromatography (HPAEC) with pulsed amperometric detection (PAD) was used to separate and quantify the major 15 oligosaccharides in human milk (Table 1). The analysis was based on previous published methods [23,24,25], but adopted to the following system to achieve optimal separation of the measured oligosaccharides. 

The high-performance anion-exchange chromatography with pulsed amperometric detection (HPAEC-PAD) system ICS-3000 (Dionex, Sunnyvale, CA, USA) was equipped with a thermostated CarboPac PA-200 column (3 × 50 mm guard column and 3 × 250 mm analytical column), an electrochemical Au detector and an Ag/AgCl reference electrode. The flow rate was 0.5 mL/min and the injection volume 20 µL. Separation was achieved using different gradient programs. For neutral oligosaccharides a constant concentration of 20 mM NaOH and a gradient with sodium acetate (NaOAc) from 0 to 25 mM at 5 to 30 min (30 °C) or 6 to 37 min (25 °C) were used. For acidic (sialylated) oligosaccharides a constant concentration of 0.1 M NaOH and a two-step gradient of NaOAc from 20 mM to 80 mM at 5 to 30 min and from 80 mM to 150 mM at 30 to 40 min were used. HPAEC was performed at both 30 and 40 °C for acidic oligosaccharides, to achieve optimal separation [26]. The different HMOs were identified by comparing their retention times to those of known milk oligosaccharide standards that were analysed in each run. All oligosaccharide standards were from Dextra Laboratories (Reading, UK), except for DSLNT, 3SL and 6SL, which were from Sigma-Aldrich. The oligosaccharide concentrations were calculated from the individual HMOs peak areas in relation to the area of the internal standard (galacturonic acid for acidic and stachyose for neutral oligosaccharides). The results were further corrected for the response factor of each individual oligosaccharide, as determined by the analysis of standards with known concentrations.

### 2.5. Statistical Analyses

The primary outcome and other continuous variables with skewed distributions were analysed with Mann-Whitney U test, while *t*-test for independent samples were employed for continuous variables with normal distributions. Fisher’s exact test was used for categorical outcome variables. Baseline characteristics were summarized by means and standard deviations (SDs) for continuous data and counts and percentages for categorical data. Primary and secondary outcome variables were summarized by means and SD or medians with IQR for continuous data and counts and percentages for categorical data. The analysis of similarity (ANOSIM) was applied to test if HMOs composition was more similar within the same mother over time than between mothers [27]. ANOSIM provides an R-value where R close to 1 indicates dissimilarity and R close to 0 indicates even distribution (of high and low ranks) within and between groups. As a complement to ANOSIM, permutation analysis of variance (PERMANOVA) was also applied to test if HMO composition differed among the mothers and over time. The distribution of HMOs composition across mothers and sampling times were observed by Non-metric Multi-dimensional Scaling (NMDS) plots. NMDS maps the pair-wise (dis)similarity of ranked distances to a k-dimensional ordination space, where the distance between objects corresponds to their (dis)similarity [28]. The statistical discrimination was at a significance level of 0.05. No adjustments for multiple comparisons were made for those outcomes, for which there were separate hypotheses, such as NEC, sepsis, and growth rate parameters. All statistical analyses were performed using IBM SPSS Statistics software, version 25 (IBM Corp, Armonk, NY, USA), except for Shannon diversity index, ANOSIM, PERMANOVA, and NMDS that were performed in R version 3.3.0 [29].

## 3. Results

### 3.1. Description of the Study Population

Breast milk samples were collected from 91 mothers to 106 infants at 14 (78 mothers to 89 infants) and 28 (71 mothers to 83 infants) days after delivery and at PMW 36 + 0 (56 mothers to 65 infants). Baseline characteristics of the ELBW infants are displayed in Table 2.

### 3.2. Development of the HMOs Over Time

**Figure 1:**
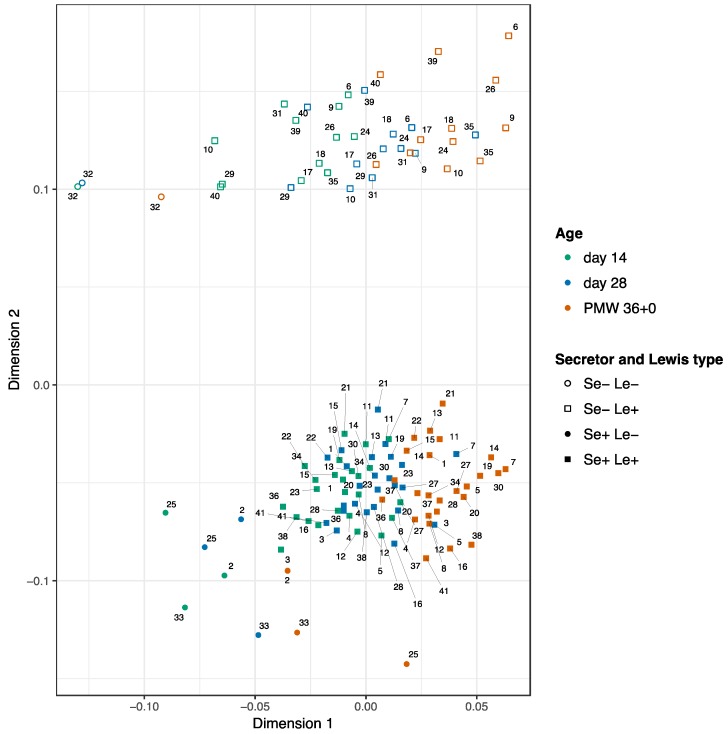
Non-metric multidemension scaling (NMDS) plot of the HMO composition among the 41 mothers with samples from all three time points. Each mother is indicated by a number from 1 to 41. PMW 36 + 0: postmenstrual week 36 + 0. Stress level: 0.08

Concentrations of the 15 HMOs in breast milk samples from 14 and 28 days after delivery and at PMW 36 + 0 are displayed in Table 1. ANOSIM analyses including the 41 cases with samples from all three time points indicated a higher variability in HMOs composition between mothers (R-value = 0.04; *p* = 0.01) than within the same mother over time (R-value = 0.7; *p* = 0.001). The HMO composition was clearly separated by the secretor status (R-value = 0.9; *p* = 0.001), which was also revealed in the NMDS plot (Figure 1). The PERMANOVA analyses showed that the secretor status explained 63% (*p* = 0.001) of the variance in HMO composition, the mother explained 26% (*p* = 0.001) and sampling time point only 0.07% (*p* = 0.001). When adjusting for secretor status, the mother explained 70% (*p* = 0.001), while the sampling time point explained 17% (*p* = 0.001) of the variance for both, secretor-positive, and secretor-negative cases.

### 3.3. Clinical Outcomes in Relation to the HMO Levels

**Figure 2:**
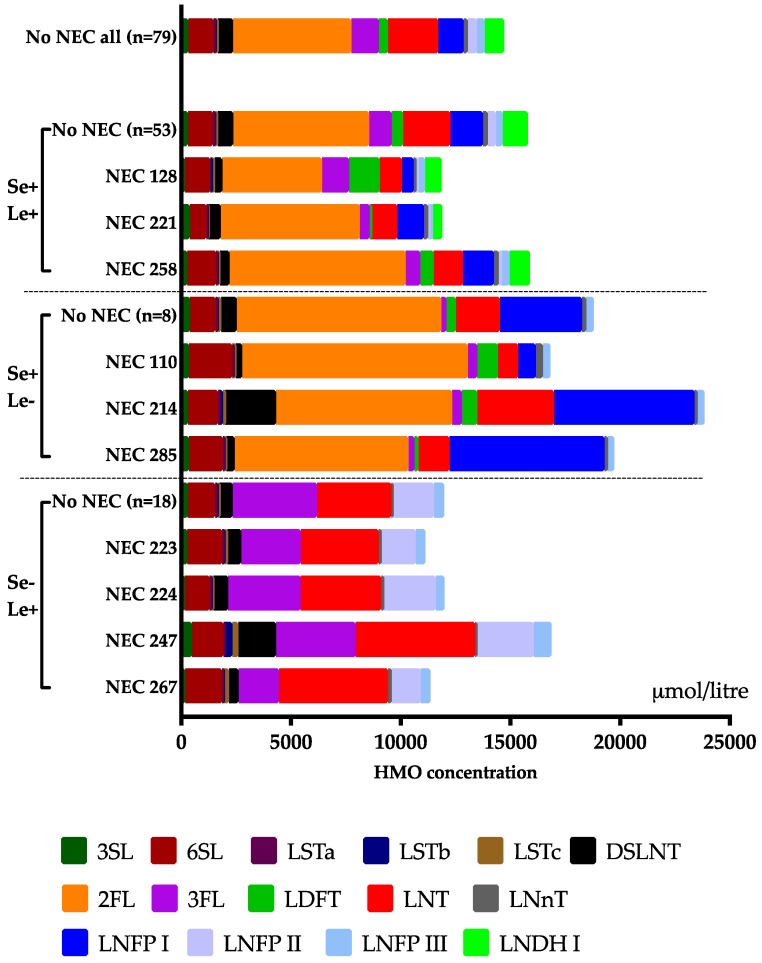
Human milk oligosaccharide (HMO) content in the first breast milk sample from mothers of all NEC-cases, stratified according to secretor (Se) and Lewis (Le) status, and median HMO levels in milk samples from day 14 from the non-NEC cases of the respective Se/Le groups

**Figure 3:**
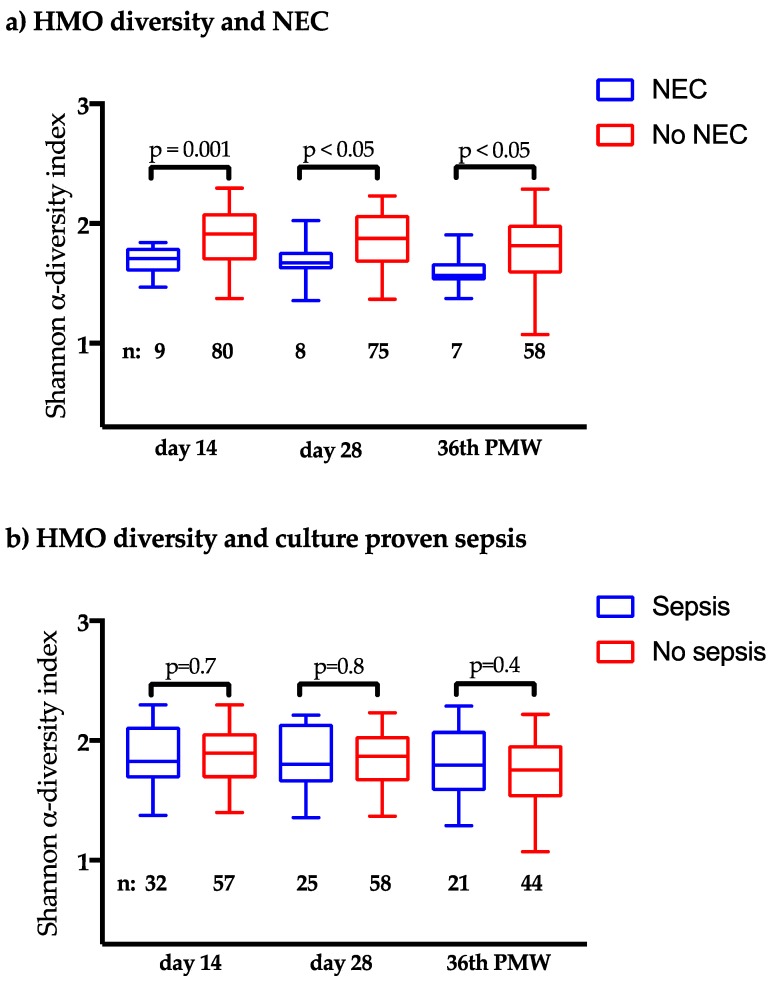
Human milk oligosaccharide (HMO) diversity in breast milk samples and the incidence of NEC (**a**) and culture proven sepsis (**b**). Boxes indicate 25^th^, 50^th^ and 75^th^ percentiles; whiskers indicate min and max values. *t*-test for independent samples was used to compare means. N indicates the number of patients in each group. PMW=postmenstrual week.

The HMO composition at day 14 in each NEC case is shown in Figure 2, and the levels of the 15 HMOs in samples from mothers to infants who did and did not developed NEC and culture-proven sepsis, respectively, are displayed in Table 3 and 4 (day 14), S1 and S2 (day 28), and S3 and S4 (PMW 36 + 0). NEC development was associated with low levels of the neutral oligosaccharide LNDH I at all three sampling time points. This HMO is only produced by mothers that are both secretor and Lewis-positive (Table 1). NEC development was also associated with low levels of LSTa and LNnT at 28 days. The diversity of all 15 HMOs was lower in all three samples from mothers to NEC cases as compared to non-NEC cases (Figure 3a), while there was no difference between NEC and non-NEC cases in the total content of HMO in the samples (Table 3, Tables S1 and S3). There was also a trend towards less NEC among infants to secretor and Lewis-positive mothers (Figure S1). The HMO diversity in NEC and non-NEC cases stratified by the different secretor groups is displayed in Figure S2. Male gender was significantly more common among the NEC-cases (Table 2), but there were no differences in HMO diversity in breast milk from mothers of singleton boys and girls (*p* = 0.43 at 14 days, *p* = 0.76 at 28 days, and *p* = 0.84 at postmenstrual week 36 + 0). The incidence of NEC was not associated with probiotic supplementation (Table 2).

High levels of 6SL at 14 days (Table 4) and low levels of 3SL and at 28 days of life (Table S2) were significantly associated with the development of culture-proven sepsis, although HMO diversity was not (Figure 3b).

**Figure 4:**
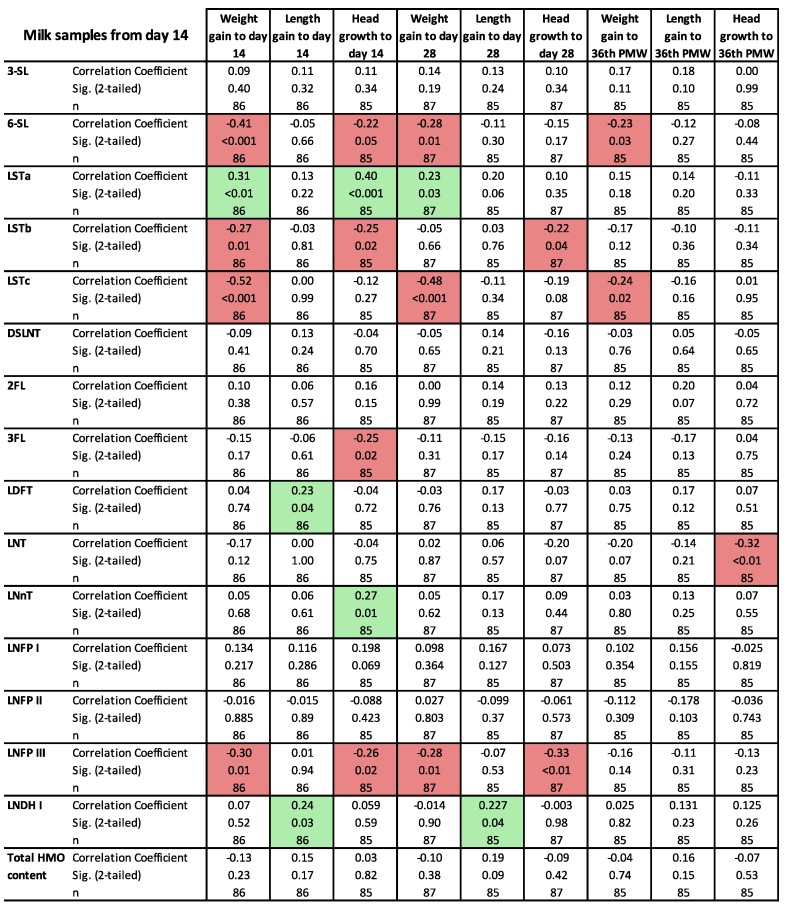
Correlations between concentrations of individual human milk oligosaccharide (HMO) (micromol/litre) in milk samples from day 14 and infant growth (change in z-score from birth to measurement on the 14^th^ or 28^th^ day of life or at 36 postmenstrual week (PMW), analysed using Spearman´s rho correlation coefficient. Significant correlations are highlighted with green for positive and red for negative correlations.

The correlation between growth indices and the 15 HMOs are displayed in Figure 4 (14-days sample) Figure S3 (28-days sample) and Figure S4 (PMW-36 + 0 sample). There were many statistically significant but weak correlations, but some of them were consistent throughout the neonatal period. The sialylated oligosaccharide LSTa correlated positively to the growth of weight and length at all three time points and also to head growth at day 14 of life, while the sialylated oligosaccharides 6SL, LSTb and LSTc correlated negatively to weight and head growth at 14 and 28 days of life.

## 4. Discussion

This study in preterm ELBW infants supports the hypothesis that HMO composition in breast milk affect the risk of developing NEC, which is consistent with animal models [14,15,30] and a previous human trial in preterm VLBW infants [16]. The main finding in the present trial was the low diversity of the 15 dominant HMOs in infants developing NEC, which has not been reported in any of the previous trials [14,15,16]. The study could not confirm the preventive effect of the sialylated HMO DSLNT on NEC found in previous studies [14,15,16], but instead NEC was associated with low levels of LNDH I, which is a fucosylated HMO that can only be produced by mothers that are both secretor and Lewis positive.

The HMO composition reflects the mother´s secretor status and Lewis blood group and depends on genetic variation causing different expression of fucosyltransferases. As a consequence, secretor-negative and Lewis-negative mothers cannot produce some of the HMOs. One of them, 2FL, is the most abundant HMO in milk from secretor-positive mothers and has been associated with preventive effects on both NEC [15,30] and infections [31]. There was a trend of lower NEC in infants to secretor and Lewis-positive, as compared to negative, mothers in the present study, and the difference in HMO diversity between NEC and non-NEC cases remained when the material was stratified to include only secretor and Lewis-positive mothers, but disappeared when only secretor and Lewis negative individuals were included. Interestingly, the two infants developing NEC despite high levels of DSLNT had received breast milk from either a secretor-negative or a Lewis-negative mother.

Diversity is crucial for resilience in biological systems [32]. For instance, high microbial diversity in the gut has been associated with reduced risk of developing NEC in preterm infants [33] and asthma in school-age children [34]. The fact that such a high variety of HMOs are produced in human breast milk also suggests that the diversity and composition of the HMOs is more important than single HMOs. However, this does not preclude a preventive effect of administration of a single HMO such as DSNLT [14,15], 2FL [30], or LNDH I in preterm infants, but such an effect still needs to be confirmed in a randomised-controlled trial. The different findings in the human trial on VLBW infants by Autran et al. [16] with a significant correlation between NEC and low DSLNT, but not with total diversity, might be explained by cohort differences: The infants in the present trial were much smaller and immature than in the previous trial [16]. We speculate that the diversity is more important in ELBW than in VLBW infants. Additionally, HMO composition may vary between different countries. Interestingly, Swedish mothers had the lowest DSLNT levels in a previous worldwide study [17].

Human milk oligosaccharides have the potential to influence many of the mechanisms attributed to NEC development such as the immaturity of the immune system, regulation of the microvascular circulation, gut motility, reduced intestinal epithelial barrier function, and aberrant gut microbiota [35,36]. Thus, HMOs have been shown to reduce neutrophil infiltration and activation in vitro [37] and stimulate anti-inflammatory responses in the intestinal epithelium ex vivo [12,13]. The secretor-dependent 2FL modulated CD14 expression in human enterocytes and attenuated LPS-induced inflammation ex vivo [38], and also attenuated the severity of experimental NEC by enhancing mesenteric perfusion in the neonatal intestine via endothelial nitric oxide activation in a murine model [30]. In a murine ex vivo model on gut motility, fucosylated, but not sialylated HMOs diminished colon motor contractions [39]. Moreover, HMOs have structural homology to many cell surface glycans and thus act as decoys by binding to luminal bacteria that are then unable to bind to the surface of the enterocyte [40]. They can mimic carbohydrate-binding motifs of certain enteric pathogens, such as enteropathogenic *E. coli* by acting as a receptor decoy, and prevent adhesion to the apical surface of enterocytes [11]. Low microbial diversity [33] and high abundance of Proteobacteria and *Enterobacteriaceae,* and low abundance of Bacteroidetes have been related to the development of NEC [41]. Bifidobacteria and Bacteroidetes species become dominant intestinal bacteria in healthy breast-fed term infants due to their ability to digest and utilise HMOs via specific glycosidases, while most pathogenic *Enterobacteriaceae* lack these enzymes and are unable to utilize HMOs as a food source [42]. Interestingly, preterm infants to secretor-negative mothers have been shown to have increased abundance of Proteobacteria [43].

Group B Streptococcus (GBS) is the most common pathogen causing neonatal sepsis. The neutral oligosaccharide LNT inhibited growth of GBS in vitro [19], and infants to Lewis-positive mothers were less colonised with GBS in a study in The Gambia [31]. Our study did not confirm these findings.

Breastfeeding has been associated with weight development in infants [44]. There were many but quite weak correlations between growth indices and single HMO in the present study, although some of our findings were consistent throughout the neonatal period. The sialylated LSTa correlated positively to the growth of weight, length and head circumference, which is consistent with a Malawian study in which sialylated HMOs were less abundant in breast milk from mothers to severely stunted infants [18]. Supplementation with sialylated bovine milk oligosaccharide also increased growth in a gnotobiotic animal model [18]. However, not all sialylated HMOs seem to increase growth. In a Gambian study, the sialylated oligosaccharides 3SL and LSTc were positively and negatively correlated, respectively, to weight development [45], while 6SL and LSTc were negatively associated to weight gain in the present study.

Our study has many strengths. It is the largest trial of its kind, although the non-significant associations between NEC and several of the HMOs, such as DSLNT and 2FL, still might be due to insufficient statistical power. Also, the study included only ELBW infants, which is the patient group with the highest risk of developing NEC. The prospective design ensured well-controlled data and precise diagnoses, and samples were obtained longitudinally at three time points during the entire neonatal period. The breast milk sampling was standardised and included all mothers with sufficient breast milk. Fifteen of the most abundant HMOs [46] were measured with an established method, HPAEC-PAD [23,24]. A limitation of the study was the fact that no infants receiving donor milk or formula were included. Moreover, since we collected breast milk samples only at three fixed time points, the duration between sampling and use of the breast milk and the NEC and sepsis onset varied between cases, although most cases started before day 28 of life. However, the variability in HMO composition was higher between mothers than within the same mother over time, and the difference in HMO diversity and LNDH I levels were significantly associated with NEC at all three sampling points. These findings implicate that the duration between the sampling and NEC onset was of minor importance. Like the previous human trial [16], the analyses purely focused on HMO concentrations and not on the absolute HMO amounts received, which depended on the amount milk the infant received. Male gender was significantly more common among the NEC-cases, but there were no differences in HMO diversity in breast milk from mothers of singleton boys and girls. No other potential confounder that we measured differently between NEC and non-NEC cases. However, this does not preclude that some other factors could have influenced the result. A randomized-controlled intervention is needed to prove causality.

## 5. Conclusions

Our results suggest that the HMO composition in breast milk may be an important factor explaining why exclusively breast milk fed ELBW infants develop NEC. Low HMO diversity and low levels of the HMO LNDH I was associated with NEC development in ELBW infants. A preventive effect of supplementation with single or multiple HMO compounds still needs to be confirmed in future randomised-controlled trials.

## Figures and Tables

**Table 1:** Median human milk oligosaccharides (HMO) levels (µmol/L) in breast milk from mothers to extremely low birth weight (ELBW) infants during the neonatal period.

Name	Type	Secreted by	Day 14 *n* = 78 Median (IQR)	Day 28 *n* = 71 Median (IQR)	36th PMW *N* = 56 Median (IQR)
**3′-sialyllactose (3SL)**	Sialylated	All	328 (255–395)	292 (226–336)	207 (161–280)
**6′-sialyllactose (6SL)**	Sialylated	All	1197 (935–1587)	821 (602–1019)	291 (191–488)
**Sialyl-lacto-N-tetraose a (LSTa)**	Sialylated	All	9 (5–14)	4 (3–8)	3 (2–5)
**Sialyl-lacto-N-tetraose b (LSTb)**	Sialylated	All	69 (42–127)	102 (45–142)	75 (33–108)
**Sialyl-lacto-N-neotetraosec (LSTc)**	Sialylated	All	130 (86–197)	79 (48–107)	20 (12–34)
**Disialyl-lacto-N-tetraose (DSLNT)**	Sialylated	All	657 (475–1049)	669 (394–910)	389 (206–516)
**2′-fucosyllactose (2FL)**	Neutral	Se+	5390 (0–7383)	4720 (0–7072)	4379 (1964–5840)
**3′-fucosyllactose (3FL)**	Neutral	All	1241 (569–2005)	1486 (795–2680)	1803 (1061–3003)
**Lacto-difucotetraose (LDFT)**	Neutral	Se+	394 (0–685)	388 (0–717)	466 (38–645)
**Lacto-N-tetraose (LNT)**	Neutral	All	2294 (1708–3138)	2205 (1640–2897)	1529 (1112–2189)
**Lacto-N-neotetraose (LNnT)**	Neutral	All	180 (96–263)	151 (86–220)	155 (90–271)
**Lacto-N-fucopentaose I (LNFP I)**	Neutral	Se+	1163 (0–1852)	819 (0–1714)	536 (95–1126)
**Lacto-N-fucopentaose II (LNFP II)**	Neutral	Le+	401 (147–948)	384 (152–826)	324 (177–685)
**Lacto-N-fucopentaose III (LNFP III)**	Neutral	All	362 (261–496)	402 (301–503)	423 (320–518)
**Lacto-N-difucohexaose I (LNDH I)**	Neutral	Se+ Le+	652 (0–1176)	726 (0–1173)	454 (0–968)
**Σ analyzed HMO**			15770 (12694–17393)	14992 (12803–17655)	11676 (10157–13703)

PMW = Postmenstrual week. IQR = Interquartile range. HMO = human milk oligosaccharides.

**Table 2:** Background characteristics in extremely low birth weight (ELBW) infants with and without necrotising enterocolitis (NEC) during the neonatal period.

	No NEC (*n* = 96)	NEC (*n* = 10)	*p* *
Gestational age, weeks + days, mean (SD)	25 + 4 (9 days)	25 + 1 (9 days)	0.3
Birth weight, g, mean (SD)	749 (136)	681 (123)	0.1
Birth weight zscore, mean (SD)	−1.1 (1.2)	−1.4 (1.6)	0.6
Birth length, cm, mean (SD)	32.7 (2.5)	32.0 (2.2)	0.4
Birth length z-score, mean (SD)	−1.5 (1.8)	−1.4 (2.0)	0.9
Birth head circumference, cm, mean (SD)	23.0 (1.5)	22.8 (1.2)	0.5
Birth head circumference z-score, mean (SD)	−0.8 (0.8)	−0.6 (0.7)	0.4
Small for gestational age, *n* (%)	22 (23)	2 (20)	0.8
Caesarean section, *n* (%)	62 (65)	6 (60)	0.8
Apgar score 5 min, mean (SD)	6.2 (2.6)	7.0 (2.4)	0.3
Apgar score 10 min, mean (SD)	7.8 (1.9)	8.4 (1.6)	0.3
Male, *n* (%)	49 (51)	9 (90)	<0.01
Infants from multiple pregnancy, *n* (%)	35 (36)	4 (40)	0.8
Maternal smoking, *n* (%)	2 (2)	1 (10)	0.5
Maternal preeclampsia, *n* (%)	10 (10)	1 (10)	1.0
Preterm premature rupture of membranes, *n* (%)	30 (30)	4 (40)	0.6
Maternal chorioamnionitis, *n* (%)	20 (21)	3 (30)	0.6
Maternal prepartal antibiotics, *n* (%)	51 (53)	5 (50)	0.9
Prenatal steroids, *n* (%)	94 (81)	10 (100)	0.2
Received surfactant, *n* (%)	78 (81)	9 (90)	0.4
Antibiotics during first week, *n* (%)	95 (99)	10 (100)	1.0
Antibiotics during second week, *n* (%)	76 (79)	10 (100)	0.2
Probiotic supplementation, *n* (%)	50 (52)	5 (50)	1.0
Patent ductus arteriosus treated, *n* (%)	70 (73)	7 (70)	1.0

* *t*-test for independent samples to compare means. Fisher’s exact test to compare proportions.

**Table 3:** Comparison of human milk oligosaccharide (HMO) concentrations (µmol/L) in milk samples from day 14 to infants who developed or did not develop necrotising enterocolitis (NEC).

	Secreted by	NEC (*n* = 9) Median (IQR)		No NEC (*n* = 80) Median (IQR)		p *
**3SL**	All	318	(231–376)	321	(254–395)	0.8
**6SL**	All	1437	(1220–1635)	1159	(919–1585)	0.2
**LSTa**	All	9	(7–17)	10	(5–14)	0.6
**LSTb**	All	49	(29–144)	68	(42–128)	0.4
**LSTc**	All	138	(103–196)	130	(85–198)	0.7
**DSLNT**	All	572	(401–1193)	674	(498–1038)	0.4
**2FL**	Se+	6331	(0–8026)	5390	(3374–7223)	0.9
**3FL**	All	675	(436–3004)	1255	(575–1841)	0.6
**LDFT**	Se+	45	(0–651)	410	(28–686)	0.2
**LNT**	All	3507	(1277–4320)	2294	(1710–2990)	0.7
**LNnT**	All	129	(90–208)	187	(111–273)	0.2
**LNFP I**	Se+	797	(0–3903)	1165	(441–1789)	0.5
**LNFP II**	Le+	124	(0–1996)	413	(191–880)	0.6
**LNFP III**	All	363	(332–437)	351	(267–526)	0.8
**LNDH I**	Se+ Le+	0	(0–213)	882	(0–1279)	<0.01
**Σ analyzed HMO**		15889	(11623–18311)	15770	(13405–17274)	0.8

* Mann Whitney *U*-test for independent samples used to compare distributions.

**Table 4:** Comparison of human milk oligosaccharide (HMO) concentrations (µmol/L) in milk samples from day 14 to infants who developed or did not develop culture-proven sepsis.

	Secretedby	Sepsis (*n* = 32) Median (IQR)		No Sepsis (*n* = 57) Median (IQR)		p *
**3-SL**	All	283	(236–336)	331	(268–399)	0.2
**6-SL**	All	1313	(1088–1647)	1142	(876–1503)	<0.05
**LSTa**	All	7	(4–12)	11	(7–14)	0.1
**LSTb**	All	62	(34–154)	72	(42–114)	0.5
**LSTc**	All	134	(102–174)	127	(79–199)	0.8
**DSLNT**	All	622	(426–941)	644	(502–1093)	0.2
**2FL**	Se+	4829	(0–7674)	5944	(3429–7467)	0.3
**3FL**	All	1367	(595–2983)	1227	(524–1833)	0.4
**LDFT**	Se+	386	(0–664)	404	(22–690)	0.7
**LNT**	All	2459	(1481–3630)	2194	(1768–2710)	0.5
**LNnT**	All	202	(100–273)	177	(110–257)	0.9
**LNFP I**	Se+	957	(0–1625)	1490	(475–2042)	0.2
**LNFP II**	Le+	494	(171–1348)	372	(123–749)	0.3
**LNFP III**	All	363	(269–468)	351	(255–531)	0.8
**LNDH I**	Se+ Le+	632	(0–1194)	821	(0–1231)	0.5
**Σ analyzed HMO **		15723	(13137–17176)	15972	(13008–17512)	0.8

* Mann Whitney *U*-test for independent samples used to compare distributions.

## Supplementary Materials

The following are available online at http://www.mdpi.com/2072-6643/10/10/1556/s1, Figure S1: NEC among infants to secretor- and Lewis-positive mothers, Figure S2: The HMO diversity in NEC and non-NEC cases stratified by the different secretor groups, Figure S3: The correlation between growth indices and the 15 HMOs at day28, Figure S4: The correlation between growth indices and the 15 HMOs atPMW 36 + 0, Table S1: HMOs at day 28 from mothers to infants who did and did not developed NEC, Table S2: HMOs at day 28 from mothers to infants who did and did not culture-proven sepsis, Table S3: HMOs at PMW 36 + 0 from mothers to infants who did and did not developed NEC. Table S4: HMOs at PMW from mothers to infants who did and did not developed culture-proven sepsis.
