# Supplementary material for: Low Diversity of Human Milk Oligosaccharides is Associated with Necrotising Enterocolitis in Extremely Low Birth Weight Infants
Source: Nutrients. 2018 Oct 20;10(10):1556. doi: 10.3390/nu10101556 (PMC6213888; doi:10.3390/nu10101556)
Supplement: Supplementary file 1 [file nutrients-10-01556-s001.zip › Supplementary figures and tables 180925/Figure S4 HMO PMW 36 growth 180922.pdf]

Milk samples from 36th postmenstrual week		Weight gain to 36th PMW	Length gain to 36th PMW	Head growth to 36th PMW
**3-SL**	Correlation Coefficient	0.21	0.21	0.06
	Sig. (2-tailed)	0.09	0.10	0.64
	n	65	65	65
**6-SL**	Correlation Coefficient	-0.21	0.04	0.06
	Sig. (2-tailed)	0.09	0.74	0.65
	n	65	65	65
**LSTa**	Correlation Coefficient	0.27	0.23	-0.13
	Sig. (2-tailed)	0.03	0.07	0.32
	n	65	65	65
**LSTb**	Correlation Coefficient	-0.18	0.02	-0.05
	Sig. (2-tailed)	0.15	0.88	0.71
	n	65	65	65
**LSTc**	Correlation Coefficient	-0.34	-0.25	0.14
	Sig. (2-tailed)	0.01	0.05	0.27
	n	65	65	65
**DSLNT**	Correlation Coefficient	-0.08	0.13	-0.05
	Sig. (2-tailed)	0.51	0.29	0.67
	n	65	65	65
**2FL**	Correlation Coefficient	0.23	0.15	0.20
	Sig. (2-tailed)	0.07	0.24	0.11
	n	65	65	65
**3FL**	Correlation Coefficient	-0.25	-0.20	-0.17
	Sig. (2-tailed)	0.05	0.12	0.18
	n	65	65	65
**LDFT**	Correlation Coefficient	-0.07	-0.03	-0.02
	Sig. (2-tailed)	0.59	0.83	0.89
	n	65	65	65
**LNT**	Correlation Coefficient	-0.12	0.01	-0.12
	Sig. (2-tailed)	0.33	0.95	0.36
	n	65	65	65
**LNnT**	Correlation Coefficient	0.01	-0.13	-0.01
	Sig. (2-tailed)	0.93	0.32	0.97
	n	65	65	65
**LNFP I**	Correlation Coefficient	0.18	0.16	0.12
	Sig. (2-tailed)	0.15	0.20	0.36
	n	65	65	65
**LNFP II**	Correlation Coefficient	-0.21	-0.13	-0.11
	Sig. (2-tailed)	0.10	0.31	0.37
	n	65	65	65
**LNFP III**	Correlation Coefficient	0.02	-0.11	-0.02
	Sig. (2-tailed)	0.87	0.38	0.86
	n	65	65	65
**LNDH I**	Correlation Coefficient	-0.10	0.06	0.02
	Sig. (2-tailed)	0.42	0.63	0.87
	n	65	65	65
**Total HMO content**	Correlation Coefficient	0.11	0.13	0.11
	Sig. (2-tailed)	0.36	0.32	0.40
	n	65	65	65

Figure S4. Correlations between concentrations of individual HMO (micromol/litre) in milk samples from the 36th postmenstrual week and growth (change in z-score from birth to measurement at 36 postmenstrual weeks), analyzed using Spearman's rho correlation coefficient. Significant correlations are highlighted with green for positive and red for negative correlations.
