# Supplementary material for: Low Diversity of Human Milk Oligosaccharides is Associated with Necrotising Enterocolitis in Extremely Low Birth Weight Infants
Source: Nutrients. 2018 Oct 20;10(10):1556. doi: 10.3390/nu10101556 (PMC6213888; doi:10.3390/nu10101556)
Supplement: Supplementary file 1 [file nutrients-10-01556-s001.zip › Supplementary figures and tables 180925/Table S4 sepsis PMW36 180923.docx]

| **Table S4**: Comparison of HMO concentrations (μmol/litre) in milk samples from the 36^th^ postmenstrual week to infants who developed or did not develop culture-proven sepsis. | | | | | | | |
| --- | --- | --- | --- | --- | --- | --- | --- |
|  | **Secreted**  **by** |  | **Sepsis (n=21)**  **Median (IQR)** | | **No sepsis (n=44)**  **Median (IQR)** | | ***p**** |
| **3-SL** | All |  | 202 | (143-276) | 202 | (161-271) | 0.9 |
| **6-SL** | All |  | 384 | (246-485) | 283 | (170-521) | 0.5 |
| **LSTa** | All |  | 3 | (2-6) | 3 | (2-5) | 0.5 |
| **LSTb** | All |  | 90 | (42-130) | 61 | (32-92) | 0.1 |
| **LSTc** | All |  | 20 | (14-48) | 20 | (12-33) | 0.6 |
| **DSLNT** | All |  | 403 | (205-510) | 314 | (201-516) | 0.6 |
| **2FL** | Se+ |  | 4243 | (1057-6148) | 4657 | (2240-5899) | 0.6 |
| **3FL** | All |  | 2020 | (965-3013) | 1583 | (1051-2961) | 0.7 |
| **LDFT** | Se+ |  | 450 | (107-668) | 472 | (38-645) | 1.0 |
| **LNT** | All |  | 2085 | (1220-2313) | 1485 | (1071-2109) | 0.2 |
| **LNnT** | All |  | 203 | (137-295) | 131 | (85-204) | 0.07 |
| **LNFP I** | Se+ |  | 305 | (96-1322) | 544 | (95-1001) | 0.9 |
| **LNFP II** | Le+ |  | 417 | (227-709) | 284 | (121-643) | 0.3 |
| **LNFP III** | All |  | 435 | (368-544) | 402 | (292-487) | 0.2 |
| **LNDH I** | Se+ Le+ |  | 407 | (0-1112) | 475 | (0-834) | 0.8 |
| **Σ analyzed HMO** |  |  | 11661 | (10316-14786) | 11731 | (9828-13474) | 0.6 |
| *Mann Whitney *U-*test for independent samples used to compare distributions. | | | | | | | |
