# Supplementary material for: Low Diversity of Human Milk Oligosaccharides is Associated with Necrotising Enterocolitis in Extremely Low Birth Weight Infants
Source: Nutrients. 2018 Oct 20;10(10):1556. doi: 10.3390/nu10101556 (PMC6213888; doi:10.3390/nu10101556)
Supplement: Supplementary file 1 [file nutrients-10-01556-s001.zip › Supplementary figures and tables 180925/Figure S2 secretor status diversity NEC and sepsis 180922.pdf]

**Figure S2:**

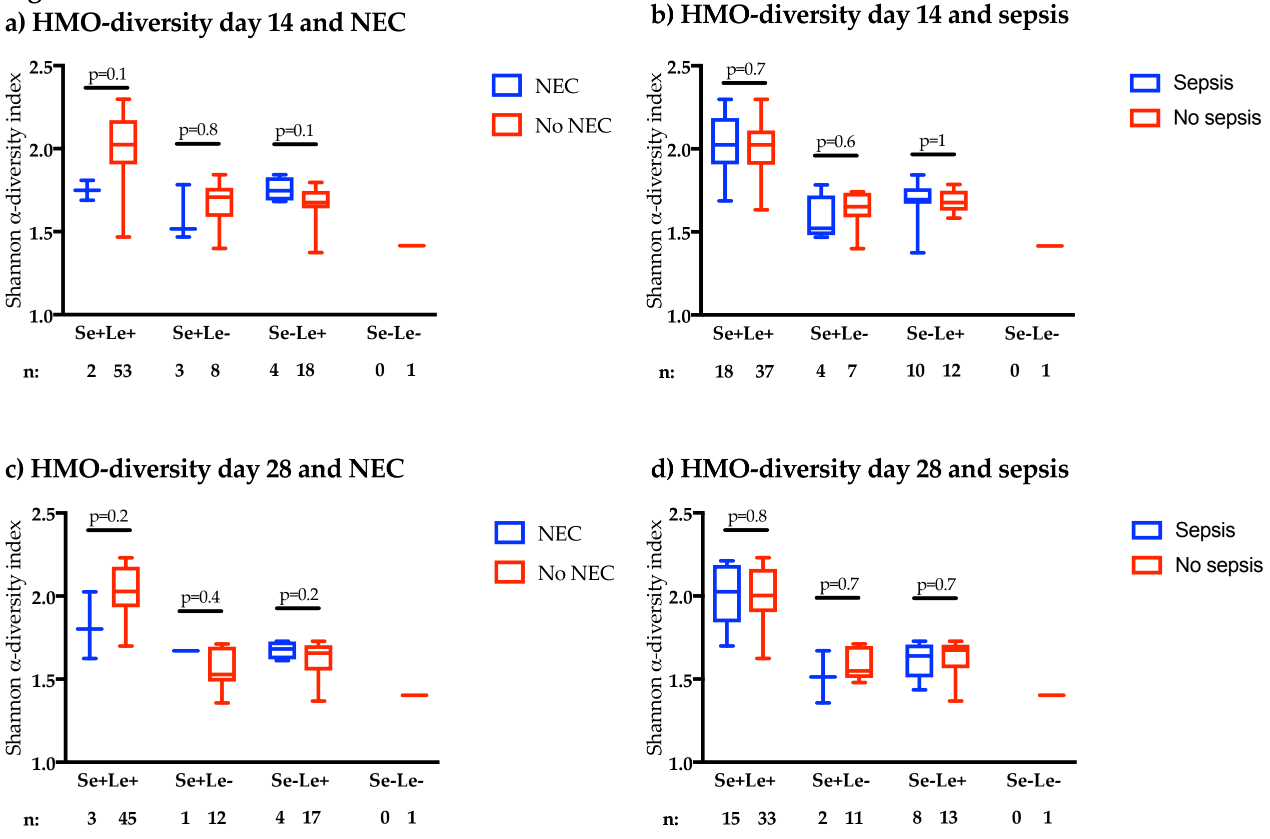


Figure S2: HMO diversity in breast milk samples from day 14 (a and b) or 28 (c and d); and incidence of NEC (a and c) and culture proven sepsis (b and d), plotted according to secretor and Lewis status of the mother. Boxes indicate 25th, 50th and 75th percentiles; whiskers indicate min and max values. *t*-test for independent samples was used to compare means.
