# Supplementary material for: Low Diversity of Human Milk Oligosaccharides is Associated with Necrotising Enterocolitis in Extremely Low Birth Weight Infants
Source: Nutrients. 2018 Oct 20;10(10):1556. doi: 10.3390/nu10101556 (PMC6213888; doi:10.3390/nu10101556)
Supplement: Supplementary file 1 [file nutrients-10-01556-s001.zip › Supplementary figures and tables 180925/Figure S3 HMO 28 growth 180922.pdf]

Milk samples from day 28		Weight gain to day 28	Length gain to day 28	Head growth to day 28	Weight gain to 36th PMW	Length gain to 36th PMW	Head growth to 36th PMW
**3-SL**	Correlation Coefficient	0.09	0.03	-0.07	0.18	0.14	0.06
	Sig. (2-tailed)	0.44	0.82	0.51	0.10	0.23	0.61
	n	83	81	83	81	81	81
**6-SL**	Correlation Coefficient	-0.33	-0.14	-0.32	-0.28	-0.11	-0.11
	Sig. (2-tailed)	<0.01	0.21	<0.01	0.01	0.35	0.34
	n	83	81	83	81	81	81
**LSTa**	Correlation Coefficient	0.24	0.34	0.12	0.30	0.37	0.16
	Sig. (2-tailed)	0.03	<0.01	0.30	0.01	<0.01	0.15
	n	83	81	83	81	81	81
**LSTb**	Correlation Coefficient	-0.24	-0.07	-0.28	-0.30	-0.16	-0.11
	Sig. (2-tailed)	0.03	0.54	0.01	0.01	0.16	0.34
	n	83	81	83	81	81	81
**LSTc**	Correlation Coefficient	-0.40	-0.21	-0.34	-0.22	-0.20	0.03
	Sig. (2-tailed)	<0.001	0.06	<0.01	0.05	0.07	0.81
	n	83	81	83	81	81	81
**DSLNT**	Correlation Coefficient	-0.15	0.06	-0.22	-0.10	0.02	0.00
	Sig. (2-tailed)	0.18	0.60	0.05	0.38	0.89	0.98
	n	83	81	83	81	81	81
**2FL**	Correlation Coefficient	0.07	0.20	0.12	0.27	0.25	0.12
	Sig. (2-tailed)	0.51	0.07	0.27	0.02	0.03	0.29
	n	83	81	83	81	81	81
**3FL**	Correlation Coefficient	-0.14	-0.19	-0.10	-0.25	-0.24	-0.08
	Sig. (2-tailed)	0.21	0.09	0.37	0.02	0.03	0.46
	n	83	81	83	81	81	81
**LDFT**	Correlation Coefficient	-0.14	0.06	-0.08	0.01	0.01	0.06
	Sig. (2-tailed)	0.21	0.60	0.47	0.92	0.92	0.61
	n	83	81	83	81	81	81
**LNT**	Correlation Coefficient	-0.22	-0.04	-0.31	-0.30	-0.11	-0.20
	Sig. (2-tailed)	0.04	0.70	0.01	0.01	0.34	0.08
	n	83	81	83	81	81	81
**LNnT**	Correlation Coefficient	0.037	0.168	0.075	0.158	0.169	0.207
	Sig. (2-tailed)	0.738	0.134	0.5	0.16	0.132	0.064
	n	83	81	83	81	81	81
**LNFP I**	Correlation Coefficient	0.08	0.26	0.08	0.25	0.28	0.13
	Sig. (2-tailed)	0.47	0.02	0.50	0.03	0.01	0.26
	n	83	81	83	81	81	81
**LNFP II**	Correlation Coefficient	-0.09	-0.15	-0.05	-0.22	-0.21	-0.07
	Sig. (2-tailed)	0.45	0.19	0.64	0.05	0.07	0.55
	n	83	81	83	81	81	81
**LNFP III**	Correlation Coefficient	-0.23	-0.06	-0.22	-0.07	-0.04	-0.02
	Sig. (2-tailed)	0.04	0.60	0.04	0.54	0.70	0.86
	n	83	81	83	81	81	81
**LNDH I**	Correlation Coefficient	-0.106	0.181	0.004	-0.028	0.092	0.11
	Sig. (2-tailed)	0.342	0.106	0.973	0.806	0.412	0.328
	n	83	81	83	81	81	81
**Total HMO**	Correlation Coefficient	-0.14	0.24	-0.18	0.02	0.17	0.07
	Sig. (2-tailed)	0.20	0.03	0.10	0.86	0.14	0.53
	n	83	81	83	81	81	81

Figure S3: Correlations between concentrations of individual HMO (micromol/litre) in milk samples from day 28 and infant growth (change in z-score from birth to measurement on the 28th day of life or at 36 postmenstrual weeks), analyzed using Spearman's rho correlation coefficient. Significant correlations are highlighted with green for positive and red for negative correlations.
