# Supplementary material for: Low Diversity of Human Milk Oligosaccharides is Associated with Necrotising Enterocolitis in Extremely Low Birth Weight Infants
Source: Nutrients. 2018 Oct 20;10(10):1556. doi: 10.3390/nu10101556 (PMC6213888; doi:10.3390/nu10101556)
Supplement: Supplementary file 1 [file nutrients-10-01556-s001.zip › Supplementary figures and tables 180925/Table S2 sepsis 28d 180923.docx]

| **Table S2**: Comparison of HMO concentrations (μmol/L) in milk samples from day 28 to infants who developed or did not develop culture-proven sepsis. | | | | | | | |
| --- | --- | --- | --- | --- | --- | --- | --- |
|  | **Secreted**  **by** |  | **Sepsis (n=25)**  **Median (IQR)** | | **No sepsis (n=58)**  **Median (IQR)** | | ***p**** |
| **3-SL** | All |  | 228 | (178-316) | 299 | (244-357) | <0.01 |
| **6-SL** | All |  | 888 | (640-1098) | 826 | (602-991) | 0.5 |
| **LSTa** | All |  | 3 | (2-9) | 5 | (3-9) | 0.4 |
| **LSTb** | All |  | 68 | (36-132) | 89 | (47-142) | 0.3 |
| **LSTc** | All |  | 86 | (39-116) | 76 | (50-107) | 0.8 |
| **DSLNT** | All |  | 472 | (324-857) | 683 | (480-927) | 0.09 |
| **2FL** | Se+ |  | 4131 | (0-6041) | 5691 | (2291-7392) | 0.06 |
| **3FL** | All |  | 1486 | (1048-3625) | 1483 | (741-2533) | 0.4 |
| **LDFT** | Se+ |  | 359 | (0-512) | 435 | (87-772) | 0.2 |
| **LNT** | All |  | 2328 | (1198-3293) | 2009 | (1562-2803) | 0.6 |
| **LNnT** | All |  | 151 | (67-244) | 152 | (93-207) | 0.8 |
| **LNFP I** | Se+ |  | 737 | (0-1495) | 999 | (118-1923) | 0.1 |
| **LNFP II** | Le+ |  | 640 | (194-937 | 345 | (107-636) | 0.1 |
| **LNFP III** | All |  | 362 | (293-457) | 416 | (311-507) | 0.4 |
| **LNDH I** | Se+ Le+ |  | 762 | (0-992) | 658 | (0-1187) | 0.9 |
| **Σ analyzed HMO** |  |  | 14144 | (12363-15745) | 15246 | (12989-19055) | 0.2 |
| *Mann Whitney *U-*test for independent samples used to compare distributions. | | | | | | | |
