# Supplementary material for: Low Diversity of Human Milk Oligosaccharides is Associated with Necrotising Enterocolitis in Extremely Low Birth Weight Infants
Source: Nutrients. 2018 Oct 20;10(10):1556. doi: 10.3390/nu10101556 (PMC6213888; doi:10.3390/nu10101556)
Supplement: Supplementary file 1 [file nutrients-10-01556-s001.zip › Supplementary figures and tables 180925/Figure S1 Secretor status NEC and sepsis 180922.pdf]

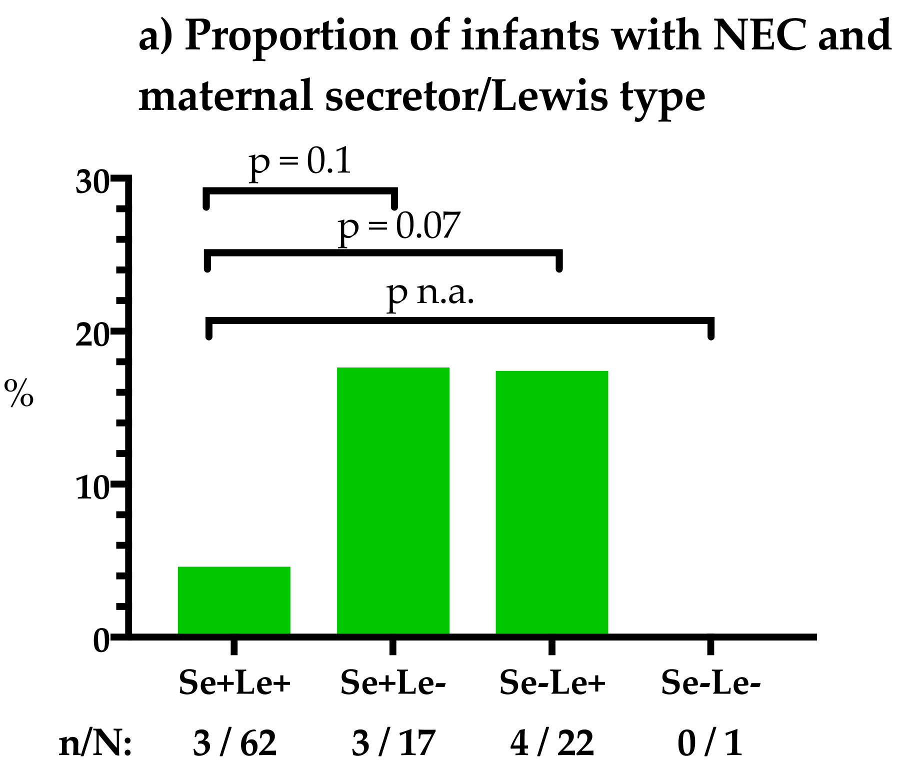

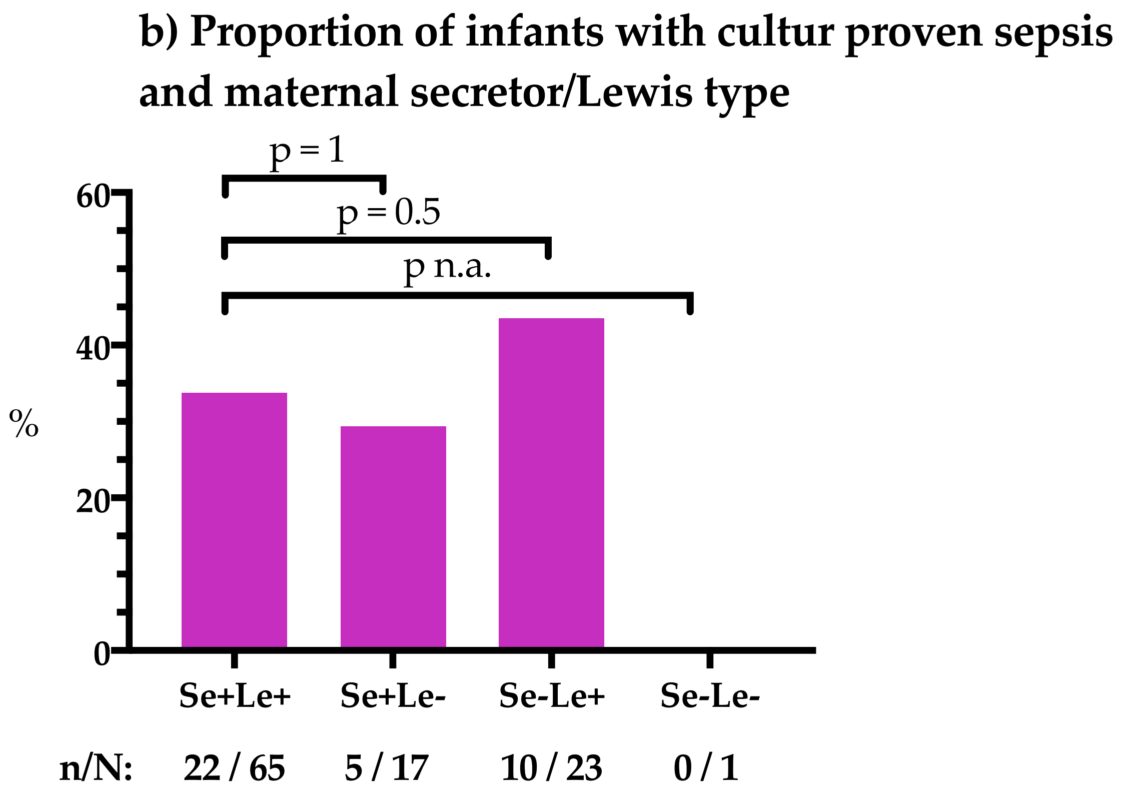

Figure S1: n/N indicates the number of infants with or without NEC (a) or culture proven sepsis (b). Fisher's exact test was used to compare the Se+Le+ group with the other groups.
