# Supplementary material for: Low Diversity of Human Milk Oligosaccharides is Associated with Necrotising Enterocolitis in Extremely Low Birth Weight Infants
Source: Nutrients. 2018 Oct 20;10(10):1556. doi: 10.3390/nu10101556 (PMC6213888; doi:10.3390/nu10101556)
Supplement: Supplementary file 1 [file nutrients-10-01556-s001.zip › Supplementary figures and tables 180925/Table S1 NEC 28d 180923.docx]

| **Table S1**: Comparison of HMO concentrations (μmol/L) in milk samples from day 28 to infants who developed or did not develop NEC. | | | | | | | |
| --- | --- | --- | --- | --- | --- | --- | --- |
|  | **Secreted**  **by** |  | **NEC (n=8)**  **Median (IQR)** | | **No NEC (n=75)**  **Median (IQR)** | | ***p**** |
| **3-SL** | All |  | 240 | (177-327) | 288 | (226-336) | 0.4 |
| **6-SL** | All |  | 969 | (644-1131) | 836 | (602-983) | 0.4 |
| **LSTa** | All |  | 3 | (2-3) | 5 | (3-10) | <0.05 |
| **LSTb** | All |  | 58 | (32-138) | 94 | (45-141) | 0.5 |
| **LSTc** | All |  | 87 | (45-128) | 78 | (49-107) | 0.8 |
| **DSLNT** | All |  | 403 | (346-653) | 697 | (394-910) | 0.2 |
| **2FL** | Se+ |  | 2275 | (0-6964) | 5151 | (2285-7150) | 0.3 |
| **3FL** | All |  | 2368 | (780-3972) | 1483 | (777-2354) | 0.5 |
| **LDFT** | Se+ |  | 150 | (0-656) | 405 | (116-717) | 0.2 |
| **LNT** | All |  | 2067 | (1018-3217) | 2018 | (1563-2860) | 0.7 |
| **LNnT** | All |  | 70 | (16-144) | 157 | (95-227) | <0.05 |
| **LNFP I** | Se+ |  | 268 | (0-986) | 976 | (157-1774) | 0.07 |
| **LNFP II** | Le+ |  | 563 | (62-1969) | 379 | (152-746) | 0.8 |
| **LNFP III** | All |  | 389 | (307-492) | 412 | (301-503) | 0.9 |
| **LNDH I** | Se+ Le+ |  | 0 | (0-662) | 762 | (0-1211) | 0.07 |
| **Σ analyzed HMO** |  |  | 13686 | (10934-15094) | 15162 | (13051-18881) | 0.2 |
| *Mann Whitney *U-*test for independent samples used to compare distributions. | | | | | | | |
