# Supplementary material for: Low Diversity of Human Milk Oligosaccharides is Associated with Necrotising Enterocolitis in Extremely Low Birth Weight Infants
Source: Nutrients. 2018 Oct 20;10(10):1556. doi: 10.3390/nu10101556 (PMC6213888; doi:10.3390/nu10101556)
Supplement: Supplementary file 1 [file nutrients-10-01556-s001.zip › Supplementary figures and tables 180925/Table S3 NEC PMW36 180923.docx]

| **Table S3**: Comparison of HMO concentrations (μmol/L) in milk samples from the 36^th^ postmenstrual week to infants who developed or did not develop NEC. | | | | | | | |
| --- | --- | --- | --- | --- | --- | --- | --- |
|  | **Secreted**  **by** |  | **NEC (n=7)**  **Median (IQR)** | | **No NEC (n=58)**  **Median (IQR)** | | ***p**** |
| **3-SL** | All |  | 202 | (137-270) | 203 | (160-275) | 0.6 |
| **6-SL** | All |  | 432 | (241-525) | 292 | (190-495) | 0.6 |
| **LSTa** | All |  | 3 | (2-4) | 3 | (2-5) | 0.7 |
| **LSTb** | All |  | 57 | (27-102) | 75 | (32-111) | 0.6 |
| **LSTc** | All |  | 20 | (6-20 | 23 | (14-35) | 0.2 |
| **DSLNT** | All |  | 237 | (194-438) | 376 | (203-525) | 0.5 |
| **2FL** | Se+ |  | 4243 | (0-6584) | 4458 | (2590-5954) | 0.6 |
| **3FL** | All |  | 2640 | (414-4400) | 1803 | (1052-2770) | 0.6 |
| **LDFT** | Se+ |  | 239 | (0-450) | 487 | (198-647) | 0.2 |
| **LNT** | All |  | 1515 | (1122-2113) | 1507 | (1080-2275) | 1.0 |
| **LNnT** | All |  | 119 | (67-203) | 176 | (92-275) | 0.4 |
| **LNFP I** | Se+ |  | 264 | (0-1166) | 536 | (162-1148) | 0.5 |
| **LNFP II** | Le+ |  | 268 | (0-1065) | 312 | (183-665) | 1.0 |
| **LNFP III** | All |  | 435 | (321-505) | 428 | (319-508) | 0.9 |
| **LNDH I** | Se+ Le+ |  | 0 | (0-444) | 490 | (0-993) | <0.05 |
| **Σ analyzed HMO** |  |  | 10646 | (8727-11661) | 11772 | (10241-14005) | 0.3 |
| *Mann Whitney *U-*test for independent samples used to compare distributions. | | | | | | | |
